# The effect of calcium palmitate on bacteria associated with infant gut microbiota

**DOI:** 10.1002/mbo3.1187

**Published:** 2021-05-15

**Authors:** Lu Wang, Gabriela Bravo‐Ruiseco, Randolph Happe, Tao He, Jan Maarten van Dijl, Hermie J. M. Harmsen

**Affiliations:** ^1^ Department of Medical Microbiology University of Groningen University Medical Center Groningen Groningen The Netherlands; ^2^ Ausnutria B.V Zwolle The Netherlands

**Keywords:** calcium palmitate, cell structure, *Faecalibacterium prausnitzii*, gut microbiota, infant nutrition

## Abstract

Gut microbiota development in formula‐fed and breast‐fed infants is known to differ. This could relate to the usage of unmodified vegetable oil instead of mammalian fat in infant formula (IF), causing the enhanced formation of the poorly soluble soap calcium palmitate (CP) in the infant's gut. Here we investigate *in vitro* the possible influence of CP on the infant gut bacteria. The growth of several bacterial species dominant in the infant's gut was analyzed by culturing in media with CP. *Faecalibacterium prausnitzii* as a sensitive representative was analyzed in detail by scanning transmission electron microscopy, membrane staining, gas chromatography, and microbial fuel cell experiments. Of all bacteria tested, the growth of several bifidobacteria and *F. prausnitzii* was reduced at 0.01 mg/ml CP, *Bifidobacterium infantis* stopped growing completely. CP reduced the cell envelope thickness of *F. prausnitzii*, disturbed the cell membrane fatty acids and function of membrane proteins involved in electron transport. CP inhibited the growth of bifidobacteria and faecalibacteria. This suggests that modification of fat in IF may benefit the development of the gut microbiota in formula‐fed infants by supporting the colonization of important beneficial bacteria in early life. Future clinical studies are needed to confirm this.

## Graphical abstract

The use of unmodified vegetable oil instead of mammalian fat in infant formula causes the enhanced formation of the poorly soluble soap calcium palmitate in the infant's gut. This study shows that calcium palmitate inhibits bacterial growth, reduces the bacterial cell envelope thickness, and disturbs the function of membrane proteins involved in electron transport.

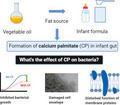

**Abbreviations**
CFU: Colony forming units
CP: Calcium palmitate
EET: Extracellular electron transfer
FISH: Fluorescence *in situ* hybridization
IF: Infant formula
MFC: Microbial Fuel Cell
SSL: Sodium stearoyl lactylate
STEM: Scanning transmission electron microscope

## 1 INTRODUCTION

Breast milk is the recommended nutrition for newborns. However, for newborns who cannot receive breast milk, infant formula (IF) is a good alternative. Importantly, the type of nutrition is known to influence the gut microbiota composition, and an aberrant microbiota development has been associated with diseases, like asthma and type 1 diabetes (Harmsen & Goffau, 2016). It was previously observed that breastfed infants display a more complex bifidobacterial diversity and, after weaning, a higher abundance of butyrate‐producing bacteria than formula‐fed infants (Stewart et al., 2018; Roger et al., 2010; Yasmin et al., 2017). This focuses interest on the differences between breast milk and IF. One of the differences is the presence of human milk oligosaccharides in breast milk which strongly stimulate the bifidobacterial population in the infant's gut (Moore & Townsend, 2019). Nowadays, IF is often supplemented with various oligosaccharides to mimic the effects of breast milk on the gut microbiota.

Another difference is that during the production of IF, milk fat is often replaced by vegetable oils. Both fats are triglycerides, that is esters of fatty acids and glycerol, but there are important differences (Havlicekova et al., 2015). In breast milk triglycerides, ~60% of the major saturated fatty acid, palmitic acid, is esterified at the sn‐2 (or β‐) position (Innis et al., 1994). In contrast, triglycerides in standard IFs have only 13% of the palmitic acid esterified at the sn‐2 position, while most palmitic acid is esterified at the sn‐1 and sn‐3 positions. Yet, some IFs with modified vegetable oil contain triglycerides with ~50% palmitic acid at the sn‐2 position (Yaron et al., 2013). Upon digestion, palmitic acid esterified at the sn‐2 position will be absorbed in the form of mono‐acylglycerol. However, palmitic acids at the sn‐1 and sn‐3 position are released into the gut and can then bind minerals like calcium to form insoluble soaps, especially calcium palmitate (CP). Such soaps can cause a hard stool, and lower the calcium and fatty acids absorption (Manios et al., 2020; Havlicekova et al., 2015). Changed stool consistency suggests that CP formation might influence the gut microbiota. Thus, it was proposed that sn‐2 esterified palmitic acid in IFs may influence the composition of the infant gut microbiota, especially the levels of lactobacilli and bifidobacteria (Yaron et al., 2013). In turn, this might influence late‐colonizing butyrate‐producing bacteria, like *Faecalibacterium prausnitzii*, which is regarded as a potential probiotic because of its anti‐inflammatory properties (Goffau et al., 2013; Sokol et al., 2008).

Little was thus far known about the effects of CP on bacteria colonizing the gut of infants. In the present study, we selected a panel of infant gut bacteria based on their described abundance and important role in gut microbiota development. We aimed at investigating the effect of CP on the growth characteristics of these selected bacteria *in vitro*, including early‐colonizing bifidobacteria and Bacteroides spp., and the late‐colonizing *F. prausnitzii*. Furthermore, we investigated how interactions between bifidobacteria, *F. prausnitzii*, and *Bacteroides thetaiotaomicron* are influenced by CP.

## 2 EXPERIMENTAL SECTION

### 2.1 Bacterial strains

Bacterial strains used in this study were obtained from culture collections (ATCC, DSMZ, NIZO) and our local strain collection (Dept. of Medical Microbiology, UMCG, Groningen, Netherlands (MMB)). Based on the infant gut microbiota composition (Pannaraj et al., 2017; Yang et al., 2019), the following bacterial strains were selected: *F. prausnitzii* A2‐165 (DSM 17677); *F. prausnitzii* ATCC 27768; *F. prausnitzii* HTF‐F (DSM 26943); *Bifidobacterium longum* spp. *longum* MMB‐01 (referred to as *B. longum*); *Bifidobacterium longum* spp. *infantis* ATCC 15697 (referred to as *B. infantis*); *Bifidobacterium breve* MMB‐ENR0374; *Bifidobacterium bifidum* MMB‐02; *Bifidobacterium dentidum* ATCC 27678; *Bifidobacterium adolescentis* NIZO B659; *Bacteroides fragilis* DSM2151; *Bacteroides thetaiotaomicron* ATCC 29741; *Lactobacillus acidophilus* DSM 20079; *Ruminococcus gnavus* MMB‐ENR0435; *Collinsella aerofaciens* MMB‐03; and *Escherichia coli* ATCC 29522.

### 2.2 Growth conditions

Strains were inoculated in YCFAG medium (Lopez‐Siles et al., 2012) and incubated under anaerobic conditions (80% N_2_, 12% CO_2_, and 8% H_2_) at 37°C. To measure the effects of CP on bacterial growth, the YCFAG medium was supplemented with different concentrations of CP. To this end, 1.0 g CP (Cayman Chemical) was dissolved into 20 ml of propionic acid (Sigma). Subsequently, the dissolved CP was filtered and added to a sterile YCFAG medium.

### 2.3 Influence of CP on bacterial growth in mono‐ and co‐cultures

For monocultures, 50 μl overnight culture of each strain was used to inoculate 5 ml of YCFAG medium with different concentrations of CP. In co‐culture experiments, two combinations of bacterial strains (*F. prausnitzii* and *B. longum* spp., or *F. prausnitzii* and *B. thetaiotaomicron*) were used to inoculate 5 ml CP‐supplemented YCFAG media with equal numbers of colony‐forming units (CFU, 1 × 10^7^ cells) of *F. prausnitzii*, *B. longum* spp. and/or *B. thetaiotaomicron* from overnight monocultures. Growth was monitored spectrophotometrically by measuring the optical density at 600 nm (OD_600_). All growth experiments were performed in triplicate.

### 2.4 Quantification of bacteria in co‐culture experiments by fluorescent *in situ* hybridization (FISH)

Samples of co‐cultures were collected at different time points after inoculation. FISH was performed according to the procedure described by Harmsen (Harmsen et al., 2002). Details of the experimental procedure are presented in the Appendix.

### 2.5 Staining of *F. prausnitzii* with FM 4‐64

*Faecalibacterium prausnitzii* A2‐165 was cultured for 16 h at 37°C in YCFAG medium without or with either 0.003, 0.006, 0.01, or 0.02 mg/ml CP. Culture aliquots of 100 µl were collected and centrifuged at 8000 *g* for 4 min. Cell pellets were incubated with 200 μl FM 4‐64 membrane dye solution (5 μg/ml) for 10 min at room temperature. Then 10 μl of cell suspension was mounted on a slide coated with a 2% agarose pad, and the slides were kept in the dark for 15 min before imaging with a Leica epifluorescence microscope equipped with a Nikon EOS500 camera. The fluorescence intensity was measured for 25 cells per micrograph using ImageJ software (Version 1.51n; National Institutes of Health, USA).

### 2.6 Fatty acid composition of bacterial cell membranes

*Faecalibacterium prausnitzii* A2‐165 was grown for 16 h in YCFAG medium without or with either 0.003 or 0.01 mg/ml CP, and YCFAG without propionic acid followed by a 2‐h treatment with propionic acid or 0.03 mg/ml CP. The fatty acid composition was measured by gas chromatography as described by Muskiet (Muskiet et al., 1983). For details, see the Appendix.

### 2.7 Scanning transmission electron microscopy (STEM)

To investigate cell structure changes in *F. prausnitzii* A2‐165 without or with either 0.003 or 0.01 mg/ml CP, and YCFAG without propionic acid followed by a 2‐h treatment with 0.03 mg/ml CP, STEM images were taken using a Zeiss Supra55 SEM equipped with an external scan generator (ATLAS, Fibics, Canada). Large area scans enabled the analysis of many bacteria within one data set. Sample preparation was based on a protocol described by Silva (Silva et al., 2014) with some modifications. For details, refer to the Appendix.

### 2.8 Microbial fuel cell (MFC) experiments

*Faecalibacterium prausnitzii* A2‐165 was grown for 16 h in YCFAG, followed by 2‐h incubation with 0, 0.003, 0.01 or 0.03 mg/ml CP. The MFC experiments were conducted as previously described by Khan (Khan, Browne, et al., 2012). For details, refer to the Appendix.

### 2.9 Statistical analyses

Statistical analyses were performed using GraphPad Prism version 5 (GraphPad Prism, San Diego, CA, USA). Unpaired *t*‐tests (two‐tailed) were performed to assess significance. *P*‐values <0.05 were regarded as significant.

## 3 RESULTS

### 3.1 Effects of CP on the growth of infant gut bacteria

**FIGURE 1:**
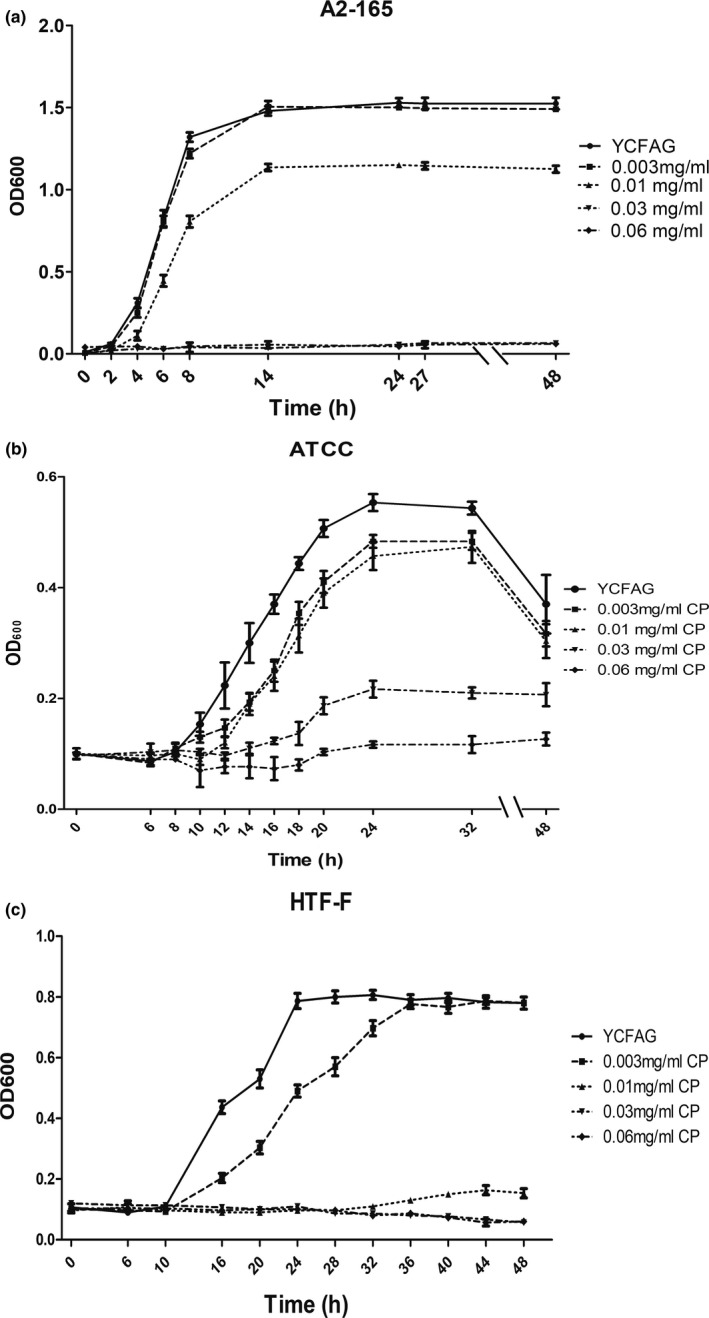
Effect of calcium palmitate (CP) on the growth of (a) *F. prausnitzii* A2‐165; (b) *F. prausnitzii* ATCC 27768; (c) *F. prausnitzii* HTF‐F (DSM 26943)

Breast‐fed and formula‐fed infants display a different gut microbiota composition, which may relate to CP formation (Yao et al., 2014). To investigate how CP could influence the gut microbiota, *in vitro* growth experiments were performed with a representative panel of dominant bacteria from the infant gut (Table 1). Figure 1 shows growth curves recorded for *F. prausnitzii* at different CP concentrations. Growth of *F. prausnitzii* A2‐165 (Figure 1a) was inhibited by 0.01 mg/ml CP, and no growth was observed at higher CP concentrations; growth of *F. prausnitzii* ATCC 27768 (Figure 1b) and *F. prausnitzii* HTF‐F (Figure 1c) was inhibited by 0.003 mg/ml CP, and no growth was observed at 0.06 mg/ml. Likewise, growth of *B. breve* and *B. bifidum* was inhibited at a CP concentration of 0.01 mg/ml, while at 0.03 mg/ml CP these bacteria did not grow at all (Table 1). Growth of *B. longum* was also affected at 0.01 mg/ml CP, but this bacterium stopped growing at 0.06 mg/ml CP. In contrast, the growth of *B. infantis* already stopped at 0.01 mg/ml CP, indicating that this bacterium is more sensitive to CP than other bifidobacteria tested. Growth of *B. thetaiotaomicron* only stopped at 0.06 mg/ml CP, whereas *C. aerofaciens*, *E. coli*, *L. acidophilus*, *B. dentidum*, *B. adolescentis*, and *R. gnavus* did even grow at 0.06 mg/ml CP (Table 1). These data show that the growth of the various infant gut bacteria is differently affected by CP.

**TABLE 1:** Effect of calcium palmitate on the growth^a^ of dominant infant gut bacteria

Species	Concentrations of calcium palmitate (mg/ml)				
	0	0.003	0.01	0.03	0.06
*F. prausnitzii* A2‐165	1.53 (0.02)^b^	1.50 (0.01)	1.12 (0.01)	0.05 (0.02)	0.05 (0.02)
*F. prausnitzii* ATCC 27768	0.37 (0.04)	0.32(0.02)	0.30 (0.02)	0.21 (0.02)	0.13 (0.01)
*F. prausnitzii* HTF‐F	0.78 (0.16)	0.78 (0.02)	0.15 (0.01)	0.06 (0)	0.06 (0)
*B. infantis*	1.07 (0.02)	0.90 (0.05)	0.08 (0.04)	0.05 (0.02)	0 (0)
*B. bifidum*	1.16 (0.03)	1.09 (0.04)	0.68 (0.03)	0 (0)	0 (0)
*B. longum*	1.63 (0.06)	1.41 (0.03)	0.93 (0.05)	0.61 (0.04)	0.05 (0.01)
*B. breve*	1.26 (0.05)	1.28 (0.01)	0.53 (0.05)	0.03 (0.01)	0 (0)
*B. dentidum*	1.68 (0.07)	1.63 (0.04)	1.63 (0.03)	1.60 (0.02)	1.59 (0.03)
*B. adolescentis*	1.28 (0.02)	1.22 (0.05)	1.24 (0.04)	1.24 (0.01)	1.2 (0.03)
*B. thetaiotaomicron*	1.96 (0.03)	1.73 (0.02)	1.61 (0.01)	1.08 (0.04)	0.02 (0.01)
*B. fragilis*	1.40 (0.02)	1.32 (0.02)	1.17 (0.02)	0.78 (0.03)	0.60 (0.04)
*L. acidophilus*	1.92 (0.03)	1.94 (0.02)	1.88 (0.04)	1.87 (0.05)	1.86 (0.04)
*E. coli*	1.15 (0.04)	1.09 (0.05)	1.19 (0.01)	1.15 (0.04)	1.17 (0.02)
*R. gnavus*	1.89 (0.06)	1.82 (0.02)	1.77 (0.05)	1.69 (0.03)	1.53 (0.05)
*C. aerofeaciens*	1.93 (0.03)	1.78 (0.03)	1.43 (0.02)	1.27 (0.03)	1.15 (0.04)

^a^ The bacterial growth was monitored spectrophotometrically by measuring the OD_600_.

^b^ Values (SD) are average of three OD_600_ measurements performed on different days.

### 3.2 Influence of CP on the staining of *F. prausnitzii* A2‐165 with FM 4‐64

**FIGURE 2:**
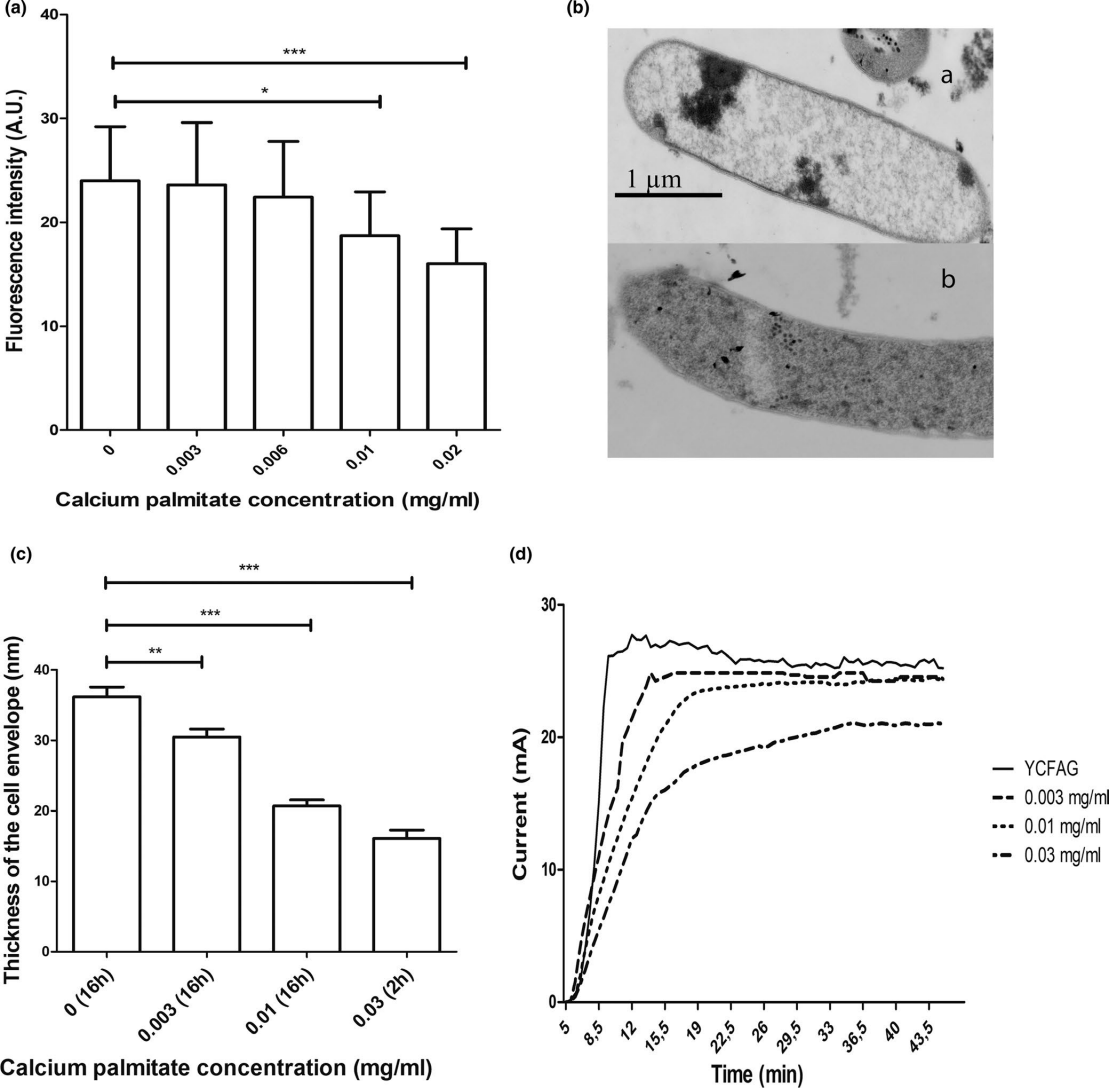
Effect of calcium palmitate (CP) on (a) Fluorescence intensity of the cell membrane of *F. prausnitzii* A2‐165 (16 h growth with different concentrations of CP) upon staining with FM‐64; (b) STEM images of *F. prausnitzii* A2‐165 (i) grown in the absence of CP, or (ii) exposed to 0.03 mg/ml CP; (c) Thickness of the cell envelope of *F. prausnitzii* A2‐165 upon growth for 16 h in the absence or presence of different concentrations of CP; (d) Current production measured by MFC of *F. prausnitzii* A2‐165 grown for 16 h and incubated with different concentrations of CP for 2 h

To evaluate whether CP affects the cell membrane integrity of *F. prausnitzii*, bacteria were stained with FM 4‐64. As *F. prausnitzii* couldn't grow at 0.03 mg/ml CP according to the growth curve results, *F. prausnitzii* was cultured for 16 h at 37°C in five different media for this experiment: YCFAG medium without or with either 0.003, 0.006, 0.01 or 0.02 mg/ml CP. Figure 2a shows that the cell membrane of *F. prausnitzii* was completely stained with FM 4‐64, independent of the presence of CP. However, the measured fluorescence intensity per cell decreased with increasing CP concentration, being significantly reduced at CP concentrations above 0.01 mg/ml (*p* = 0.023). This indicates that CP affects FM 4‐64 staining of the bacterial membrane, either due to membrane disintegration or altered membrane composition.

### 3.3 Influence of CP on the fatty acid composition of the *F. prausnitzii* A2‐165 cell membrane

**FIGURE 3:**
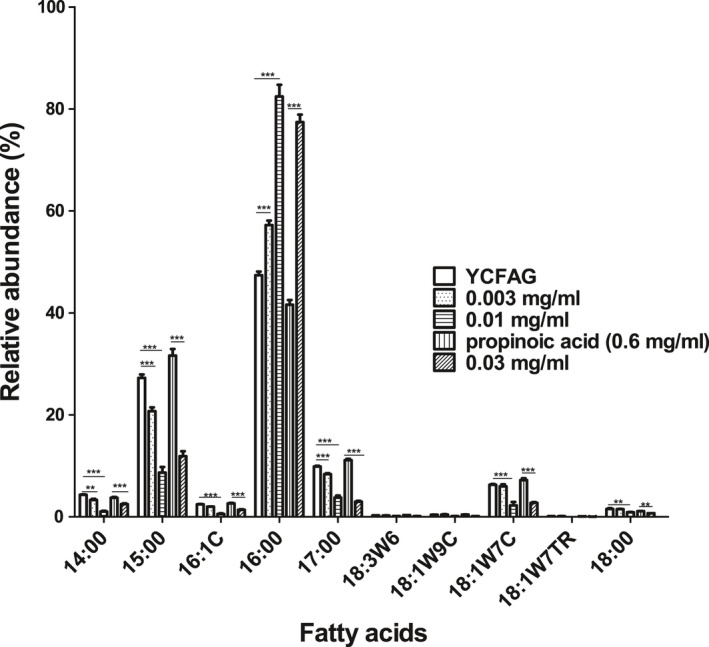
Effect of calcium palmitate (CP) on the fatty acid composition of the cell membrane of *F. prausnitzii* A2‐165 as analyzed by GC

To investigate the effects of CP on the membrane of *F. prausnitzii*, cells were grown for 16 h in YCFAG medium with either 0, 0.003, or 0.01 mg/ml CP. Since no growth was observed at 0.03 mg/ml CP, as an alternative, cells grown in YCFAG medium without propionic acid followed by 2‐h treatment with propionic acid (control) or 2‐h treatment with 0.03 mg/ml CP were included in the analysis. After membrane extraction, the fatty acid composition was analyzed by gas chromatography. Figure 3 shows that the main fatty acids in the membrane of *F. prausnitzii* grown without CP are C14:0 (myristic acid), C15:0 (pentadecylic acid), C16:0 (palmitic acid), C17:0 (margaric acid), and C18:0 (stearic acid). The levels of C14:0, C15:0, C17:0 declined upon 16‐h growth at CP concentrations increasing up to 0.01 mg/ml, whereas the C16:0 level increased. Fatty acid levels in cells treated for 2 h with 0.03 mg/ml CP were similar to the levels in cells grown for 16 h with 0.01 mg/ml CP. This shows that CP strongly affects the fatty acid composition of the *F. prausnitzii* membrane.

### 3.4 Influence of CP on cell morphology of *F. prausnitzii* A2‐165

To investigate whether CP affects the cell morphology of *F. prausnitzii*, this bacterium was grown for 16 h in YCFAG medium with either 0, 0.003, or 0.01 mg/ml CP, and YCFAG without propionic acid followed by a 2‐h treatment with 0.03 mg/ml CP. Subsequently, the cell morphology was inspected by STEM. The most prominent detected change was a reduced cell envelope thickness. Figure 2b,c show that the cell envelope thickness decreased from 36.2 nm to 16.1 nm upon increasing CP concentration from 0 to 0.03 mg/ml, respectively. When *F. prausnitzii* was treated with 0.03 mg/ml CP, a condition that stops the bacterial growth (Figure 1a), cell envelope thickness was significantly decreased (*p* < 0.0001; Figure 2c). This shows that CP affects the cell envelope structure of *F. prausnitzii*.

### 3.5 Microbial fuel cell

*Faecalibacterium prausnitzii* A2‐165 can employ an extracellular electron shuttle of flavins and thiols to transfer metabolically generated electrons to oxygen, allowing this obligate anaerobe to grow in a niche with oxygen influx from epithelial cells (Khan, Browne, et al., 2012; Khan, Duncan, et al., 2012b). This extracellular electron transport (EET) can be measured in a microbial fuel cell with glucose and riboflavin, where electrons generated by glucose fermentation are transferred via riboflavin to the anode. To determine whether CP interferes with essential processes like EET, fuel cell experiments were performed. To this end, *F. prausnitzii* A2‐165 was grown for 16 h in YCFAG, followed by 2‐h incubation with 0, 0.003, 0.01, or 0.03 mg/ml CP. Subsequently, bacteria were transferred to a fuel cell containing glucose. Irrespective of the CP treatment, 5 min after addition of riboflavin a current was measured, showing that riboflavin‐mediated EET to the anode occurred. However, the maximum current decreased with increasing CP concentration, especially when *F. prausnitzii* A2‐165 was exposed to 0.03 mg/ml CP (Figure 2d and Table 2). Also, the time necessary to reach the maximum current was increased at increasing CP concentrations. In particular, it took non‐treated bacteria ~11 min to generate a maximum current of ~26.5 mA, while it took bacteria treated with 0.03 mg/ml CP ~37 min to generate a maximum current of ~19.5 mA (Table 2). This shows that exposure of *F. prausnitzii* to CP interferes with EET and suggests an impaired function of membrane proteins needed for EET.

**TABLE 2:** Extracellular electron transport by *F. prausnitzii* A2‐165 after 16 h of growth and 2 h incubations with different concentrations of calcium palmitate

Concentration of calcium palmitate (mg/ml)	Sparklines of current production profiles	Maximum current (SD)^a^ in mA	Time for reaching the maximum current (SD) in min
0	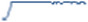	26.47 (1.28)	10.5 (0.8)
0.003	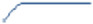	24.92 (0.06)	14.0 (0)
0.01	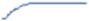	24.56 (0.39)	27.0 (1.8)
0.03	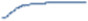	19.29 (1.77)	35.5 (1.8)

^a^ Values are average of two experiments performed on different days.

### 3.6 Interaction of *F. prausnitzii* A2‐165 with other gut bacteria in presence of CP

**FIGURE 4:**
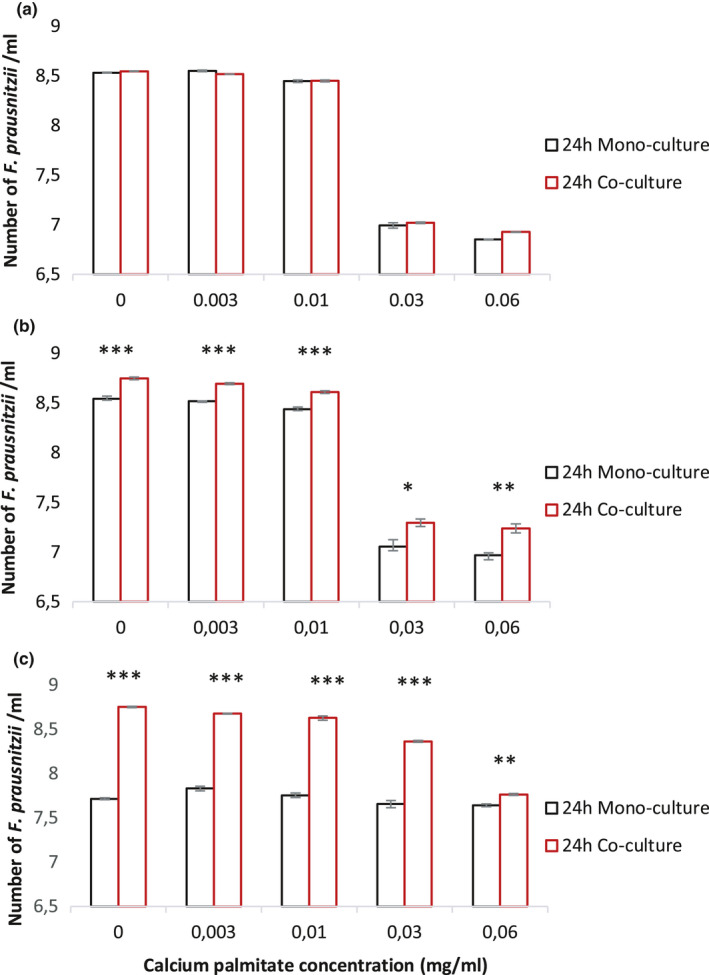
Numbers of *F. prausnitzii* A2‐165 cultured with or without other bacteria characteristic for the infant gut microbiota. The bacterial numbers were counted by FISH upon growth for 24 h in YCFAG medium without or with different concentrations of CP. *F. prausnitzii* was grown either in monoculture or co‐culture with (a) *B. longum* or (b) *B. thetaiotaomicron*. (c) Numbers of *F. prausnitzii* A2‐165 co‐cultured for 24 h with *B. thetaiotaomicron* in medium without acetate. The numbers of bacteria are presented as log10 values per ml of culture

To investigate whether other gut bacteria can help faecalibacteria to overcome the detrimental effects of CP, co‐culture experiments were performed in the YCFAG medium with CP. *B. longum* and *B. thetaiotaomicron* were used for these experiments, as they are acetate producers, while *F. prausnitzii* is an acetate consumer (Sokol et al., 2008; Duncan et al., 2002). Samples were collected at 0, 4, 8, and 24 h after inoculation. As shown by FISH, the co‐culture of *F. prausnitzii* A2‐165 with *B*. *longum* did not influence the growth of *F*. *prausnitzii*, which decreased with increasing CP concentrations, irrespective of the presence of *B*. *longum* (*p* > 0.05, Figure 4a; only numbers counted at 24 h are shown). In contrast, upon co‐culture of *F*. *prausnitzii* A2‐165 with *B. thetaiotaomicron*, the growth of *F*. *prausnitzii* was enhanced significantly. Upon *F*. *prausnitzii* exposure to 0.06 mg/ml CP, 1.1 × 10^7^ bacteria were counted in monoculture, which was similar to the bacterial number upon inoculation (1.1 × 10^7^). When co‐cultured with *B. thetaiotaomicron*, the number of *F. prausnitzii* bacteria increased to 1.8 × 10^7^ (*p* < 0.01, Figure 4b), showing a moderately beneficial effect on growth. To evaluate the importance of acetate produced by *B. thetaiotaomicron* on the growth of *F. prausnitzii* and its sensitivity to CP, mono‐ and co‐culture experiments were performed in YCFAG without acetate. As shown by FISH, acetate produced by *B. thetaiotaomicron* strongly stimulated the growth of *F. prausnitzii*, and this was even evident at 0.03 mg/ml CP (Figure 4c). This implies that other gut bacteria, like *B. thetaiotaomicron*, can mitigate detrimental effects of CP on beneficial gut microbes, like *F. prausnitzii*.

## 4 DISCUSSION

Vegetable oil blends, rich in sn‐1 and sn‐3 palmitate, are commonly used as the main fat source in IF. This may lead to increased CP concentrations in the infant's gut. Since CP is an almost insoluble soap with a water solubility of 0.03 mg/ml, it can disturb nutrient absorption in the gut of formula‐fed infants (Forsyth et al., 1999). Accordingly, it was previously reported that CP is associated with hard stools and decreased absorption of calcium and fatty acids (Litmanovitz et al., 2013). Our present study is the first to show that CP inhibits *in vitro* growth of various prominent infant gut bacteria. We report also that the growth of several bifidobacteria, such as *B. infantis*, *B. breve*, and *B. bifidum*, as well as three different strains of *F. prausnitzii*, is inhibited by CP even at low concentrations of 0.003 or 0.01 mg/ml. In contrast, the growth of other gut microbes, like *E. coli* and *B. thetaiotaomicron*, is not affected by CP.

The exact CP concentration in the infant's gut is unknown (Jandacek, 1991). Digestion of dietary fat occurs on emulsified triglyceride droplets with lipase splitting interfacial triglycerides into monoglyceride and free fatty acid. Local high concentrations of free medium‐chain and branched‐chain fatty acids may, however, prevent precipitation of calcium soaps. Other factors, like bile salts and soap solubilization by low‐melting fatty acids, may enhance the absorption of calcium and fat (Jandacek, 1991). We approximated the CP amount based on palmitic acid and calcium concentrations previously determined. For breast‐fed infants, concentrations of sn‐1/3 palmitic acid varied from 16 to 48 mg/g dry stool weight, and the calcium concentration was ~20 mg/g. In contrast, for formula‐fed infants, 72–187 mg/g palmitic acid and 32 mg/g calcium were reported (Bar‐Yoseph et al., 2016; Nowacki et al., 2014; Yao et al., 2014). Based on these numbers and stool water contents of ~73%, we estimate the maximum CP amount in the gut of breast‐fed infants at ~9 mg/g wet stool weight and in formula‐fed infants ~23 mg/g (Bar‐Yoseph et al., 2016). However, breast milk fat includes higher medium‐chain and branched‐chain fatty acids, which increase CP solubility. Therefore, the CP concentration may be lower in breastfed infants' stools. Nevertheless, the estimated CP amounts are so high that most CP will precipitate, so that the actual dissolved concentration in the infants' guts may approach the highest dissolved CP concentration in our study (0.03 mg/ml). Importantly, even a low CP concentration (0.01 mg/ml) inhibited the growth of some beneficial gut bacteria. Here, it is noteworthy that the common dietary emulsifier sodium stearoyl lactylate (SSL) has similar effects on human gut microbiota as IF. Elmén (Elmén et al., 2020) reported that a low concentration of 0.025% (w/v) of SSL already altered the human gut microbiota. Thus, our findings could help to explain the observation that *B. longum* and *B. infantis* are better colonizers of breastfed infant guts, while guts of formula‐fed infants are more often colonized by *E. coli*, *Clostridium difficile*, and *B. fragilis* group members (Penders et al., 2005, 2006; Yasmin et al., 2017). Considering the different CP amounts in the guts of breast‐fed and formula‐fed infants, we hypothesize that excess formation of calcium soaps contributes to differences in gut microbiota composition in breast‐fed and formula‐fed infants. This is an innovative concept because current attempts to establish a regular gut microbiota in bottle‐fed infants rely on IF supplementation with probiotics and fructo‐, galacto‐ or human milk oligosaccharides. In contrast, our results suggest that modified fats rich in sn‐2 palmitic acid, or long‐chain (poly‐)unsaturated fatty acid may prevent CP formation, thereby also protecting the microbiota (Jandacek, 1991; Forsyth et al., 1999).

Possible mechanisms underlying the detrimental effects of CP on gut bacterial growth were investigated using *F. prausnitzii* A2‐165. Cell membrane staining, measurements of the fatty acid composition of the membrane, and imaging of bacterial morphology by STEM revealed that CP affects the integrity of *F. prausnitzii*'s cell envelope. This idea was supported by MFC experiments, showing that increasing CP concentrations decreased the bacterial capacity for EET. This demonstrates that CP affects the EET machinery, which is sufficient to explain the growth impairment of *F. prausnitzii*. However, CP could also affect other physiological processes in the cell envelope of *F. prausnitzii*, as suggested by the observed growth inhibition of bacteria that cannot perform EET, like *B. fragilis*.

Bacterial cross‐feeding influences the infant gut microbiota composition (Scott et al., 2013). In such cross‐feeding interactions, acetate is important for large‐intestinal butyrate production. Further, Rios‐Covian (Ríos‐Covián et al., 2016) reported that co‐culture with *B. adolescentis* L2‐32 stimulated the growth of *F. prausnitizii* A2‐165, which was accompanied by decreased acetate production and increased butyrate production. Our present study shows that cross‐feeding may occur between specific combinations of bacteria as the growth of *F. prausnitizii* A2‐165 was stimulated by *B. thetaiotaomicron*, but not by *B. longum*. In fact, *B. thetaiotaomicron* stimulated the growth of *F. prausnitizii* even in absence of acetate, which shows that growth stimulation and protection of *F. prausnitizii* against CP by *B. thetaiotaomicron* is based on other factors than acetate. Nevertheless, acetate is relevant since *F. prausnitzii* requires acetate for growth in monoculture. This shows that inter‐bacterial interactions are important for the growth of *F. prausnitzii* and help to overcome soap toxicity. This is reminiscent of animal studies, where the presence of *B. thetaiotaomicron* and *E. coli* influenced colonization by *F. prausnitzii* (Lopez‐Siles et al., 2017; Wrzosek et al., 2013).

Altogether, our study shows that CP inhibits the *in vitro* growth of various infant gut commensals by damaging the structure and function of their cell envelope. CP may, thus, influence the development of the post‐natal gut microbiota, thereby posing risks for the onset of gut microbiota‐associated diseases in later life. Future studies with IF including different fats are needed to investigate the potential effects of CP on the development of the gut microbiota in newborns.

## ETHICS STATEMENT

Not required.

## CONFLICTS OF INTEREST

None declared.

## AUTHOR CONTRIBUTIONS

Lu Wang: Data curation‐Lead, Formal analysis‐Lead, Investigation‐Lead, Methodology‐Lead, Visualization‐Lead, Writing‐original draft‐Lead; Gabriela Bravo‐Ruiseco: Data curation‐Lead, Formal analysis‐Lead, Investigation‐Lead, Methodology‐Lead, Visualization‐Lead, Writing‐original draft‐Lead; Randolph Happe: Conceptualization‐Equal, Funding acquisition‐Equal, Project administration‐Equal, Writing‐review and editing‐Equal; Tao He: Conceptualization‐Lead, Funding acquisition‐Lead, Project administration‐Lead, Writing‐review and editing‐Equal; Jan Maarten van Dijl: Supervision‐Lead, Writing‐review and editing‐Lead; Hermie Harmsen: Conceptualization‐Lead, Data curation‐Lead, Formal analysis‐Lead, Funding acquisition‐Lead, Investigation‐Lead, Methodology‐Lead, Project administration‐Lead, Supervision‐Lead, Writing‐review and editing‐Lead.

## Data Availability

All the data are provided in full in the result section of this paper except for the STEM data which is available at http://nanotomy.org/OA/Wang2021MBO/index.html.

## METHODS

### Quantification of bacteria in co‐culture experiments by fluorescent *in situ* hybridization (FISH)

Samples of co‐cultures were collected at different time points after inoculation. FISH was performed according to the procedure described by Harmsen (Harmsen et al., 2002). Briefly, 1 ml of each cell suspension was fixed overnight at 4°C with 3 ml 4% paraformaldehyde solution. 10 µl aliquots of the suspensions were applied to slides, dried and fixed with 96% (v/v) ethanol for 10 min and hybridized overnight at 50°C in a dark humid stove with the oligonucleotide probes Fprau645, Bif164 and Bac303 for *F. prausnitzii*, *B. longum* and *B. thetaiotaomicron*, respectively. Then, the cells were washed, rinsed, and mounted with Vecta Shield™ (Vector Lab., Burlingame, CA). Fluorescent cells were counted using a Leica DMRXA epifluorescence microscope (Leica, Wetzlar, Germany).

### Fatty acids composition of bacterial cell membranes

*Faecalibacterium prausnitzii* A2‐165 was grown for 16 h in YCFAG medium with or without CP. The fatty acid composition was measured by gas chromatography as described by Muskiet (Muskiet et al., 1983). 10 ml aliquots of the cultures were centrifuged at 10,000 *g* for 15 min at 4°C twice to remove the growth medium. Cell pellets were resuspended in buffer containing 50 mM Tris. HCl (pH 7.5) 150 mM NaCl, 5 mM MgCl_2_ and 10% glycerol and disrupted with 0.2–0.3 mm glass beads in a FastPrep tissue homogenizer in 5 cycles of 30 s at 6.5 m/s. Membranes were collected by centrifugation at 13,000 *g*. Briefly, membranes were trans‐esterified and trimethyl‐silylated with methanol‐hydrochloric acid solution and hexane. Aliquots of 2 μl were automatically injected into a Hewlett‐Packard Model 5880 gas chromatograph equipped with a Model 7672 A automatic injection system. Nonadecanoic acid (C19:0) was used as an internal standard for this experiment. Subsequently, the relative abundance of each fatty acid was calculated for cells grown in different conditions.

### Scanning transmission electron microscopy (STEM)

To investigate cell structure changes in *F. prausnitzii* A2‐165 treated with CP, STEM images were taken using Zeiss Supra55 SEM equipped with an external scan generator (ATLAS, Fibics, Canada). Large area scans enabled the analysis of many bacteria within 1 data set. Sample preparation was based on a protocol described by Silva (Silva et al., 2014) with some modifications. A 5 ml overnight culture was treated for 1 h with different concentrations of CP. 2 ml of each sample were centrifuged at 2000 *g*  for 5 min. Pellets were fixed using 2% glutaraldehyde/2% paraformaldehyde in 0.1 M sodiumcacodylate pH 7.3 and post‐fixed in 1% osmium tetroxide/1.5% potassium ferrocyanide. They were then sequentially dehydrated in increasing concentrations of ethanol. After embedding in EPON, ultra‐thin sections (80 nm) were cut and contrasted with 2% uranyl acetate in water followed by Reynolds' lead citrate. Large area scans were made using STEM and the thickness of the cell envelope of *F. prausnitzii* was measured for 25 cells per image using ImageJ (Version 1.51n; National Institutes of Health, USA).

### Microbial fuel cell (MFC) experiments

*Faecalibacterium prausnitzii* A2‐165 was grown for 16 h in YCFAG medium with or without CP or followed by 2‐h incubation with 0.03 mg/ml CP. The MFC experiments were conducted as previously described by Khan (Khan, Browne, et al., 2012). Briefly, 38 ml culture with OD_600_ of 0.8 broth were harvested at 2000 *g*  for 15 min, washed and resuspended in potassium phosphate glucose buffer pH 7, and added to a 37°C anode chamber continuously flushed with N_2_. 40 ml 100 mM potassium phosphate buffer with 50 mM potassium ferricyanide was added to the cathode chamber. After running the device for 5 min, 0.003 g vitamin B_2_ was added to the anode chamber. Data was collected after closing the circuit for 45 min using a CHI electrochemical instrument.
